# Association evidence of CCTTT repeat polymorphism in the iNOS promoter and the risk of atrial fibrillation in Taiwanese

**DOI:** 10.1038/srep42388

**Published:** 2017-02-13

**Authors:** Lung-An Hsu, Yung-Hsin Yeh, Wei-Jan Chen, Chi-Tai Kuo, Feng-Chun Tsai, Yi-Hsin Chan, Chun-Li Wang, Chi-Jen Chang, Hsin-Yi Tsai

**Affiliations:** 1 Cardiovascular Division, Chang Gung Memorial Hospital, Chang Gung University College of Medicine, Tao-Yuan, Taiwan; 2 Division of Cardiac Surgery, Chang Gung Memorial Hospital, Chang Gung University College of Medicine, Tao-Yuan, Taiwan

## Abstract

Inducible nitric oxide synthase (iNOS) plays an important role in the pathogenesis of atrial fibrillation (AF). The *iNOS* promoter has a CCTTT-repeat length polymorphism that can determine the level of gene transcription. This study enrolled 200 AF patients and 240 controls. The length of CCTTT-repeat polymorphism in the *iNOS* promoter region was examined by polymerase chain reactions, with the alleles with ≤11 repeats designated as S and alleles with ≥12 repeats designated as L alleles. AF patients carried significantly higher frequencies of the LL genotype than control subjects (40.0% versus 28.3%, *P* = 0.010). Multivariate analysis showed that the presence of LL genotype was significantly associated with AF (odds ratio: 1.87, 95% CI = 1.10–3.17, *P* = 0.021). *In vitro*, transient transfection assay in HL-1 atrial myocytes showed that the responsiveness of *iNOS* transcriptional activity to tachypacing was correlated with the length of the CCTTT-repeats. Right atrial tissues from patients with chronic AF were investigated with immunoconfocal microscopy. Patients with LL genotype exhibited greater oxidative stress and substrate remodeling in their atria than those with non-LL genotypes. Our results suggest that the *iNOS* microsatellite polymorphism may contribute to the genetic background of AF in Chinese-Taiwanese patients.

Atrial fibrillation (AF) is an abnormal heart rhythm that occurs as a consequence of atrial electrical or structural remodeling^1^. AF is frequently associated with other cardiovascular diseases such as hypertension, ischemic heart disease, valvular heart disease, and heart failure. However, 10% to 20% of patients with AF are free of concomitant diseases and do not have any known causes (so-called lone AF)^1^.

Although AF has been considered to be a non-genetic disorder, emerging evidence suggests a heritable linkage in many patients^2^,^3^. Genetic association studies have also demonstrated the significant contribution of single gene mutations and single nucleotide polymorphisms in the development of AF. For example, variations or polymorphisms in ion channel-, calcium handling protein-, fibrosis-, conduction-, and inflammation-related genes may render certain individuals susceptible to electrical or structural remodeling and eventually fibrillation in the atrium^2^,^3^.

Nitric oxide (NO) produced in the heart by nitric oxide synthase (NOS) is a highly reactive signaling molecule and an important modulator of myocardial function. NOS catalyzes the conversion of L-arginine to L-citrulline and NO, however under certain circumstances reactive oxygen species (ROS) can be formed instead of NO (uncoupling)^4^. Three NOS isoforms are found in the heart: neuronal NOS (nNOS, NOS1), endothelial NOS (eNOS, NOS3) and inducible NOS (iNOS, NOS2). nNOS and eNOS are Ca^2+^-dependent and constitutively present enzymes in distinct subcellular locations within cardiomyocytes^5^, whereas iNOS is absent in a healthy heart, however its expression is Ca^2+^-independent and induced by pro-inflammatory mediators and/or pathological states such as hypertrophy or heart failure^6^,^7^,^8^,^9^. The responsiveness of iNOS varies according to an individual’s inherited genetic profile. A (CCTTT)_n_ pentanucleotide repeat polymorphism has been identified in the promoter region of the *iNOS* gene, and possible associations with various infectious, atopic, nasal polyp, inflammatory and autoimmune diseases have been investigated^10^,^11^,^12^,^13^,^14^,^15^,^16^,^17^,^18^. Studies using *in vitro* assays have revealed that *iNOS* promoter activity increases in parallel with the number of (CCTTT)_n_ repeats, and that longer forms have higher transcription activity^16^,^17^,^18^. Nevertheless, relatively little is known about its role in predisposing individuals to AF. Since many studies have linked oxidative stress to the pathogenesis of AF^19^,^20^,^21^,^22^, the present study aims to test if rapid pacing would induce iNOS expression by the atrial myocyte, and to elucidate the association between the *iNOS* promoter microsatellite polymorphisms, the iNOS expression in atrial tissue, and the risk of AF.

## Results

### CCTTT repeat length polymorphisms in the *iNOS* promoter were associated with AF

**Figure 1:**
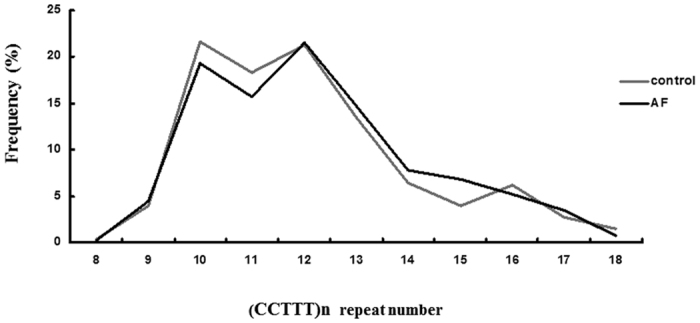
Frequency distribution of the (CCTTT)_n_ repeats in normal controls (n = 240) and AF patients (n = 200).

Table 1 presents the baseline characteristics of the 200 AF patients and 240 controls enrolled in this study. The frequencies of the classical risk factors for AF including hypertension, diabetes, and history of coronary artery disease (CAD) were slightly, but not significantly higher in the AF patients than in the controls. The concomitant use of medications was significantly different between the two groups. The allele frequencies of the (CCTTT)_n_ microsatellites in the *iNOS* promoter region in the study population are shown in Fig. 1. The distribution of allele frequencies of the (CCTTT)_n_ microsatellites in the *iNOS* promoter region was similar to that reported in a study from Japan^10^. The number of repeats ranged from 8 to 18. The difference in overall *iNOS* (CCTTT)_n_ allelic distribution between the two groups was not significant, as determined by the Kolmogorov-Smirnov test (*P* = 0.739). The distribution of the number of (CCTTT)_n_ repeats was skewed to the right, with (CCTTT)_10_ to (CCTTT)_13_ being the four most common alleles in our study cohort. Therefore, we classified the alleles into two subgroups in accordance with the best-fit grouping criteria and confirmed by sensitivity analysis (supplementary Table S1): short alleles (class S) including alleles with ≤11 repeats, and long alleles (class L) including alleles with ≥12 repeats. The subjects were then classified as having SS, SL, or LL genotypes according to each of their *iNOS* alleles. When the proportions of allele and genotype frequencies of the study population were analyzed, no significant differences were found in the distribution of allele frequencies between the control and AF groups (*P* = 0.167, Table 2). However, the distribution of genotypes was significantly different between the control and AF groups (*P* = 0.010, Table 2). The AF patients carried significantly higher frequencies of the LL genotype than the control subjects (40.0% versus 28.3%; *P* = 0.010). After adjusting for age, gender, body mass index (BMI), hypertension, diabetes, smoking, hypercholesterolemia, CAD, and the concomitant use of medications, the presence of the LL genotype remained significantly and independently associated with the risk of AF (odds ratio = 1.87, 95% confidence interval = 1.10–3.17; *P* = 0.021).

### Effect of the CCTTT repeats on *iNOS* transcriptional activity in rapidly paced HL-1 myocytes

**Figure 2:**
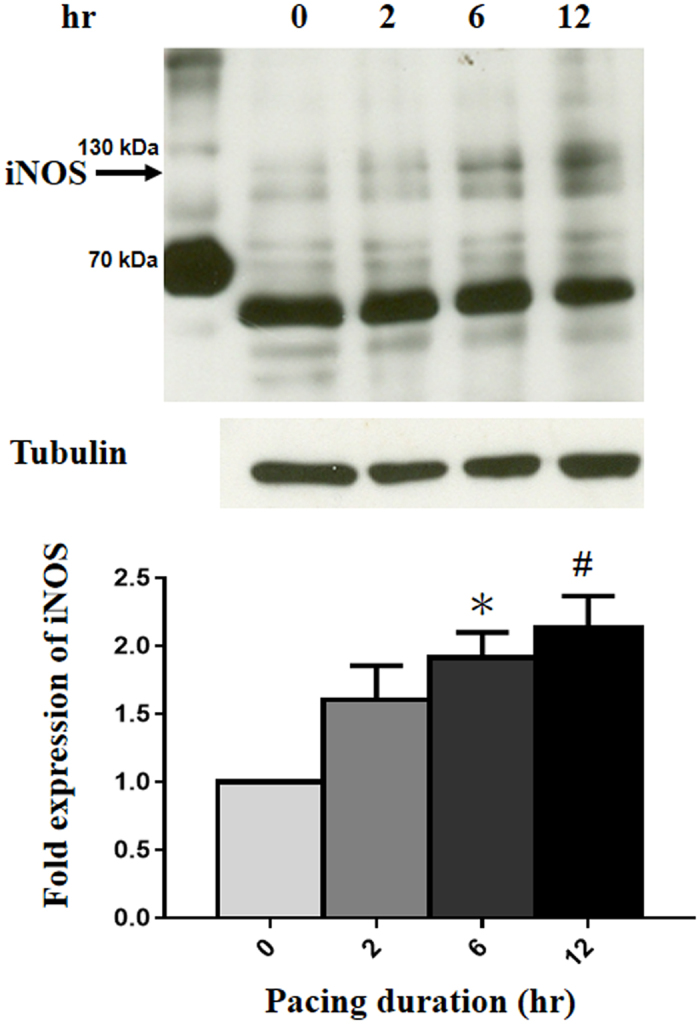
Tachypacing of HL-1 induced iNOS expression. HL-1 cells were subjected to 4 Hz pacing for the indicated times. Bottom panel: the relative expression levels of iNOS and tubulin were quantified by densitometry and normalized to the control level, which was set at 1.0. Each value represents the mean ± SE of four independent experiments. One-way ANOVA with post hoc Tukey’s tests was applied for two groups and multiple comparisons. *P = 0.027 versus the controls. ^#^*P* = 0.007 versus the controls.

**Figure 3:**
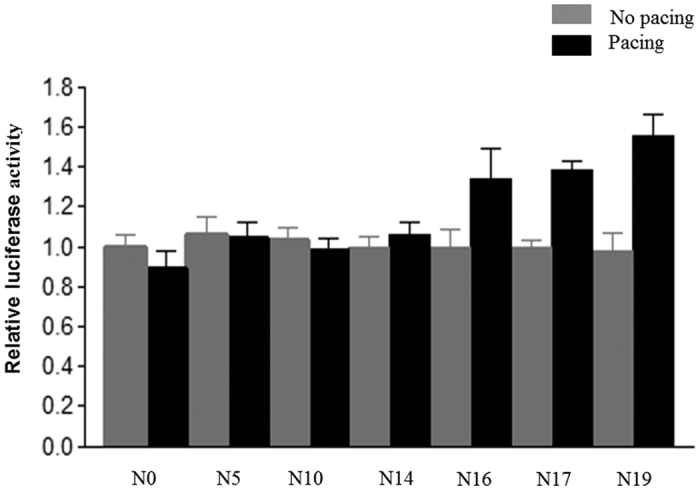
CCTTT repeats modulated the transcriptional activity of the iNOS gene and its responsiveness to rapid pacing. Luciferase activity was assayed as described in the Methods section. HL-1 cells were transfected with plasmids containing various lengths of CCTTT repeats (N) in the *iNOS* promoter for 24 hours with/without subsequent tachypacing (4 Hz) for 2 hours. Each value (mean ± SE, at least three independent experiments) is expressed as a fold change in luciferase activity relative to the control (N = 0) condition. Two-way ANOVA reported a significant effect of various lengths of CCTTT repeats (*P* = 0.005), of pacing (*P* = 0.001), and of the interaction between the main effects (*P* = 0.001).

Previous studies have demonstrated that rapid pacing of cultured HL-1 myocytes mimics the phenotypic feature of tachycardia-induced atrial remodeling *in vivo*^23^,^24^,^25^. Accordingly, we used this atrial-derived system to evaluate the effect of tachypacing *in vitro*. As shown in Fig. 2, time-dependent associations between the iNOS production of HL-1, as quantified by Western blot analysis, and the duration of atrial pacing were observed. To investigate whether the length of the CCTTT repeats affected the transcriptional regulation of the *iNOS* promoter by tachypacing, human *iNOS* promoter constructs containing different numbers of CCTTT repeats (5, 10, 14, 16, 17 and 19) were generated and transfected into HL-1 myocytes. As shown in Fig. 3, no significant differences between the baseline promoter activity and the length of the CCTTT repeats were observed in the HL-1 cells. However, there was a progressive increase in pacing-induced *iNOS* promoter activity with the plasmids contained increasing numbers of CCTTT repeats (Fig. 3). These results suggest that the length of the CCTTT repeats may modulate the responsiveness of *iNOS* transcriptional activity to tachypacing.

### Association of the length of the CCTTT repeats with atrial oxidative stress in AF tissues

**Figure 4:**
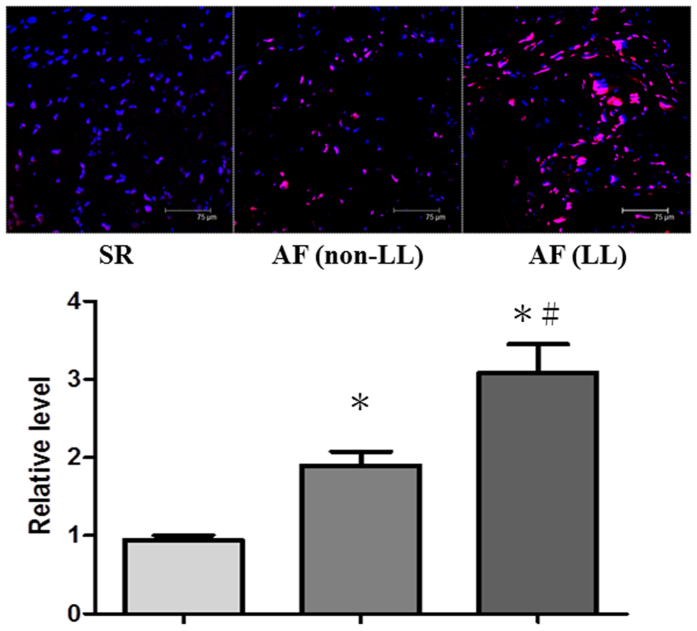
Association of the CCTTT repeat length polymorphisms with oxidative stress in AF tissues. Atrial tissues were stained with dihydroethidium to detect ROS generation as described in the Methods section. Representative confocal images show ROS production in the atria of seven AF patients (4 non-LL and 3 LL) and two controls (with sinus rhythm [SR]). Relative fluorescence density in the α-actin-expressing area was quantified (right). Data are expressed as mean ± SE. *Represents significant differences versus the controls (*P* = 0.001 for non-LL versus SR, *P* < 0.001 for LL versus SR). ^#^Represents significant differences between non-LL and LL groups (*P* < 0.001). One-way ANOVA with post hoc Tukey’s tests was applied for two groups and multiple comparisons.

**Figure 5:**
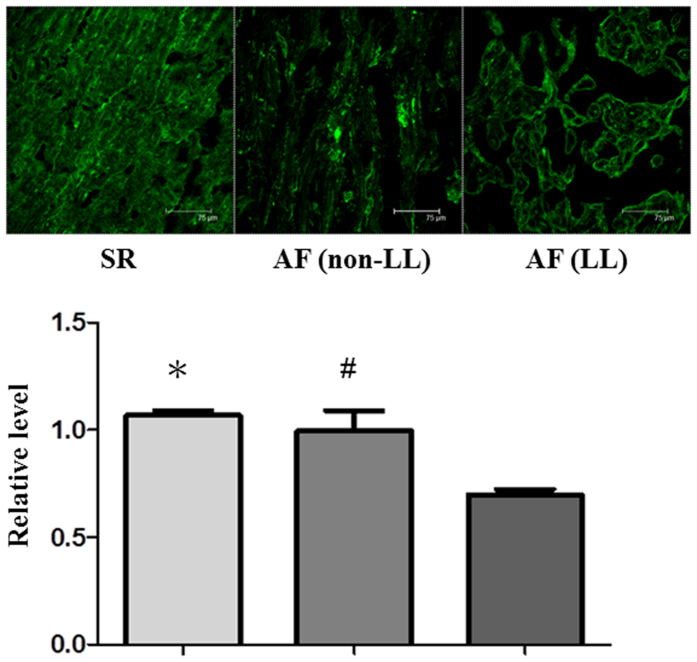
Association of the CCTTT repeat length polymorphisms with myofibril degradation in AF tissues. Representative confocal images show myosin degradation in the atria of seven AF patients (4 non-LL and 3 LL) and two controls (with sinus rhythm [SR]). Relative intensity of MHC in the α-actin-expressing area was quantified as described in the Methods section. Data are expressed as mean ± SE. *P = 0.004, SR versus LL groups. ^#^*P* = 0.004, non-LL versus LL groups. One-way ANOVA with post hoc Tukey’s tests was applied for two groups and multiple comparisons.

Our previous study showed that tachypacing promotes atrial structural remodeling, and especially myofibril degradation in atrial myocytes through increased oxidative stress^25^. To determine whether the CCTTT repeat polymorphisms affected AF-related oxidative stress and substrate remodeling, seven AF patients with four non-LL genotype and three LL genotype were chosen for comparison. Two sinus rhythm (SR) patients were designated as controls. The baseline characteristics of these nine patients are shown in the Table 3. We consistently found that oxidative stress (indicated by increased ROS generation, Fig. 4) and myofibril degradation (indicated by decreased myosin heavy chain (MHC) expression, Fig. 5) were more severe in the atria of the AF patients with LL genotype than in those with non-LL genotype and SR control. Taken together, these findings suggest that CCTTT repeat length polymorphisms may affect iNOS expression and consequent atrial structural remodeling responses in AF tissues.

## Discussion

In this study, we demonstrated a significant association between (CCTTT)_n_ pentanucleotide repeat polymorphisms in the *iNOS* gene promoter and susceptibility to AF in a Han Chinese population living in Taiwan. Individuals carrying two alleles with 12 or more repeats (high promoter activity genotype) exhibited a substantially increased risk of developing AF. Our results suggest that this functional *iNOS* gene polymorphism may contribute to the genetic background of AF.

The *iNOS* protein expression is barely detectable or undetectable in normal hearts. However, it can be highly expressed during heart failure due to stimulation via increased pro-inflammatory cytokine production such as interleukin-6 and tumor necrosis factor-alpha^4^,^6^. In humans, studies on gene expressions have shown an overexpression of *iNOS* mRNA and protein in the myocardium of patients with heart failure compared to that from unaffected controls^8^. Its expression has been detected in all four chambers, and it appears to be associated with the condition of heart failure per se rather than being related to the etiology of heart failure^8^,^9^. At low concentrations, NO may protect against apoptotic cell death induced by various stimuli, whereas it is cytotoxic at high concentrations^26^. In a recent study, Han *et al*. reported a significantly higher apoptosis index in the right atrium of patients with permanent AF compared to patients with SR, and a positive correlation with the expression of 3-nitrotyrosine (3NT; a biological marker of peroxynitrite), which was related to the increased expressions of iNOS and eNOS^27^. These findings suggest that an imbalance between iNOS and eNOS may contribute to cell apoptosis by affecting the expression of 3NT^27^. Of note, the left ventricle size and ejection fraction were comparable between the permanent AF and SR groups in Han’s study, suggesting that the induction of iNOS was not due to heart failure, but rather due to AF itself. Nishijima *et al*. recently reported increased iNOS and reduced tetrahydrobiopterin (BH_4_) expressions in failing atria in a 16-week canine tachypacing model of heart failure^28^. The authors found that this was associated with NOS uncoupling, as there was reduced NO production and increased O2• production in atrial tissue with increased atrial oxidative stress as measured by electron paramagnetic resonance spectroscopy. Furthermore, BH_4_ and L-arginine treatment reduced inducible AF and normalized the heart failure-induced shortening of the duration of left atrial myocyte action potential^28^. In a rat model of ischemic heart failure, increased atrial iNOS and Rac-1 activity was associated with atrial fibrosis and increased vulnerability to AF, both could be prevented by treatment with simvastatin which decreased myocardial oxidative stress and inflammation^29^. Taken together, these findings support that the induction of iNOS plays a role in contributing to atrial remodeling in AF.

Tachycardia-induced oxidative stress has been reported to be an important mediator in promoting and perpetuating AF^1^. Studies from our group and others have shown that oxidative stress mediates tachycardia-stimulated atrial remodeling^25^,^30^,^31^. Therefore, the responsiveness of an individual atrium to oxidative stress may determine its vulnerability to AF. Our findings support the notion that long CCTTT repeats in the *iNOS* promoter region may enhance its gene transcription in parallel with tachycardia, and subsequently increase oxidative stress and atrial remodeling. Longer CCTTT repeats have been found to increase *iNOS* transcriptional activity in several other cell types, including DLD-1 cells^16^ (human colon carcinoma cell line), human fibroblasts^17^ and HaCaT cells^18^ (human keratinocyte cell line). In this study, we did not find a significant association between baseline promoter activity and the length of the CCTTT repeats in HL-1 cells. However, we found that tachypacing-induced *iNOS* promoter activity in HL-1 cells was correlated with the length of the CCTTT repeats in the *iNOS* promoter. We further demonstrated that AF patients with LL genotype exhibited greater oxidative stress and substrate remodeling in their atria than AF patients with non-LL genotype and the SR controls, which was reflected by increased ROS generation and myofibril degradation in the atria of the AF patients homozygous for longer CCTTT repeats. It is conceivable that iNOS may locally damage the atrium by oxidative stress-induced remodeling. Taken together, our findings suggest that a combination of genetic polymorphisms and the consequent variations in NO-redox balance are involved in the pathogenesis of AF. However, we still cannot rule out the possibility that the observed association in our study is through other functional polymorphisms in linkage disequilibrium with the CCTTT repeats polymorphisms.

There are several limitations to this study. First, our findings were obtained from only one sample with a modest size. The same sample has been used in three genetic association studies before^32^,^33^,^34^. Thus, the study has a suspect validity when a multiple testing correction is stringently applied in multiple hypothesis-testing on the same sample (Bonferroni correction required *p* < 0.0125) and in the best-fit cutoff analysis. Replication in a second cohort with larger sample size would improve the strength of the analysis. However, our previous studies have different hypotheses under different *a priori* knowledge, which may not be a situation exactly like the multiple comparison scenario. Further, we and others consistently observed that the number of *iNOS* (CCTTT)n repeats above a certain threshold may be part of the causal pathway leading to the susceptibility of diseases^11^,^12^,^13^,^14^,^15^,^17^,^18^. The reason for the different (CCTTT)n cutoff points among different diseases could be that the effect size of *iNOS* (CCTTT)n repeats may not be exactly the same in different diseases and different study populations due to different environmental or genetic modifiers. Second, we did not assess the effect of the *iNOS* promoter CCTTT repeat polymorphisms on the local *iNOS* expression and 3NT levels in the atria of the patients with AF. Third, the cross-sectional nature of our study meant that we could not identify the cause and effect relationships between iNOS expression and AF-related atrial remodeling, or whether *iNOS* promoter polymorphisms could predict the future occurrence of AF in a normal individual. Finally, the study subjects were ethnically Chinese, and hence caution should be exercised when extrapolating our results to other ethnic groups.

## Conclusion

In summary, we demonstrated that the length of the polymorphism of the CCTTT repeats in the *iNOS* promoter was associated with the risk of AF and AF-related structural remodeling. These findings provide further evidence that genetic variations may influence the responsiveness to oxidative stress and the susceptibility to AF.

## Methods

### Ethics Statement

The study protocol was approved by the Human Research Ethics Committee at Chang Gung Memorial Hospital (Chang Gung Medical Foundation Institutional Review Board 101–5030B and 100–3196C1), and this study was conducted in accordance with the Declaration of Helsinki Principles. Written informed consent was obtained from each participant.

### Study population

The inclusion criteria were patients aged less than 65 years with unexplained causes of AF. Patients who had a history of hyperthyroidism, significant valvular heart disease (>grade II mitral regurgitation and/or aortic regurgitation), or congestive heart failure (left ventricular ejection fraction <50%) were excluded. A control group of patients with SR, comparable for age and gender, were recruited from individuals receiving routine health examinations. The demographic details of the study and control groups have been described elsewhere^32^,^33^,^34^.

### Clinical assessment

The presence of AF was documented by patient history, serial electrocardiograms (ECGs), and/or ambulatory ECG monitoring. Transthoracic echocardiography was performed to assess left atrial and left ventricular functions and to detect significant valvular diseases. Left atrial enlargement and left ventricular dysfunction were defined as a diameter >40 mm and ejection fraction <50%, respectively. Hypertension was defined as blood pressure ≥140/90 mmHg and/or the use of antihypertensive medication. Definitions of hypercholesterolemia and diabetes mellitus were in accordance with the third report of the National Cholesterol Education Program and the guidelines of the American Diabetes Association, respectively.

### Genomic DNA extraction

Genomic DNA was extracted from peripheral blood leukocytes and/or tissues using a Puregene DNA Isolation Kit (Qiagen, Minneapolis, MN).

### Genotyping of *iNOS* promoter microsatellite polymorphisms

The 5′-flanking region containing (CCTTT)_n_ repeats of the *iNOS* gene was amplified by polymerase chain reaction (PCR) with a FAM-labeled sense primer, 5′-ACCCCTGGAAGCCTACAACTGCAT-3′, and an antisense primer, 5′-GCCACTGCACCCTAGCCTGTCTCA-3′, according to a published procedure^35^. The PCR products were mixed together with GenoType TAMRA DNA ladder (size range, 50–500 bp; GibcoBRL) and analyzed on an automated DNA sequencer (ABI Prism 377). The respective sizes of the (CCTTT)_n_ repeats were calculated using GeneScan Analysis software (PE Applied Biosystems). To further confirm the sizes of the (CCTTT)_n_ repeats, three PCR products were subcloned into the pCRII vector (Invitrogen), and the resulting purified plasmid DNA was subjected to sequence analysis. For quality control purposes, approximately 10% of the samples were re-genotyped in a blind fashion, and the same results were obtained.

### Human samples

Right atrial appendages were obtained from seven patients with AF and two controls with SR undergoing open heart surgery. After excision, the atrial appendages were immediately frozen in liquid nitrogen and stored at −85 °C. Subsequently, genomic DNA from each subject was sent for genotyping as described above.

### Immunohistochemical analysis

Immunohistochemical analysis was performed using α-actin, iNOS, and MHC primary antibodies (Abcam, Cambridge, MA) followed by fluorescein isothiocyanate or Cy3-conjugated secondary antibodies (Chemicon, Temecula, CA). Nuclei were visualized by DAPI staining. Myosin degradation was quantified as the area containing cytoplasmic myosin (MHC) divided by the area containing nuclei. For each analysis, at least five random fields were chosen to observe >30 myocytes. ROS in the atria were measured using a fluorescent dye, dihydroethidium, a cell-permeable indicator of ROS. The tissue samples were pre-incubated with 10 μmol/L dihydroethidium for 30 minutes at room temperature, and ROS-mediated fluorescence was observed under a confocal microscope (Leica TCS SP2, Wetzlar, Germany), with excitation at 543 nm using an argon laser and emission recorded using a long pass (>600 nm) filter set to acquire two-dimensional images (512 × 512 pixels).

### Cell culture and tachypacing

HL-1 atrial myocytes were maintained in Claycomb medium as described previously^36^. HL-1 cells (≥1 × 10^6^ cells) on 4-well rectangular dishes (Nunclon, Netherlands) were placed into C-Dish 100TM-Culture Dishes (IonOptix, Milton, MA). The HL-1 cells were then subjected to field stimulation with 10-ms stimuli of 40 V intensity at 4 Hz frequency (1.5 V/cm field strength; C-Pace EP culture pacer, IonOptix)^23^,^24^,^25^. The spontaneous contraction rate was about 0.5 to 1 Hz, and a capture efficiency of >90% was confirmed by microscopic examination.

### Constructs and transfection

The *iNOS* promoters (−1557 bp to +58 bp) were amplified from genomic DNA by PCR using the forward primer 5′-GAAACGCGTGATTCTGACTCTTTCC-3′ and reverse primer 5′-CAAAGATCTGGAATGAGGCTGAGTTC-3′. The PCR products were inserted into the pGL3 basic vector (Promega, Madison, WI) at the *MluI/BglII* restriction sites. The *iNOS* promoters containing various lengths of CCTTT repeats were amplified from the genomic DNAs of the patients with AF by PCR using the forward primer 5′-CCTGGTACCCCTGGAAGCCTACAACTG-3′ and reverse primer 5′- CAAACGCGTGGCTGCAGAGAGCTA-3′ according to a previously published procedure^17^. The PCR products were then cloned into the upstream of the inserted *iNOS* gene promoter in pGL3 at *KpnI/MluI* restriction sites. For transient transfection assays, HL-1 myocytes grown to 50% to 60% confluence were transfected with the indicated plasmids using LipofectAMINE 2000 (Invitrogen) according to the manufacturer’s instructions. The transfection efficiency using this method was approximately 60%. Luciferase activities were measured with a luminometer (Luminoskan TL PMS, Thermo Lab Systems, Grand Rapids, OH).

### Statistical analysis

Continuous variables were expressed as mean ± SD and tested using a two-sample *t-*test. The chi-square test was used to examine differences in categorical variables and to compare allele and genotype frequencies. The Kolmogorov-Smirnov test was used to compare the shape of the allele frequency distribution. Sensitivity analysis was used to test all possible grouping criteria, which then classified the (CCTTT)_n_ repeats into two groups in the logistic regression models, using AF as the dependent variable and the allele groups as independent variables. Binary logistic regression analysis was used to evaluate the independent effect of genotype on the association with AF after adjusting for age, gender, BMI, hypertension, diabetes, smoking, hypercholesterolemia, CAD, and the use of concomitant medications. One-way ANOVA with post hoc Tukey’s tests were used for two groups and multiple comparisons. A two-way ANOVA was used to assess the main effects of various lengths of CCTTT repeats, tachypacing and their interaction on the *iNOS* transcriptional activity. A value of *p* < 0.05 using a two-sided test was considered to be statistically significant. All statistical analyses were performed using SPSS software version 20.0 (SPSS Inc., Chicago, IL). Missing data were approached with listwise deletion.

## Additional Information

**How to cite this article**: Hsu, L.-A. *et al*. Association evidence of CCTTT repeat polymorphism in the iNOS promoter and the risk of atrial fibrillation in Taiwanese. *Sci. Rep.* **7**, 42388; doi: 10.1038/srep42388 (2017).

**Publisher's note:** Springer Nature remains neutral with regard to jurisdictional claims in published maps and institutional affiliations.

## Supplementary Material

Supplemental Table S1

## Figures and Tables

**Table 1:** Demographic and clinical characteristics of the study population.

	Controls (n = 240)	AF patients (n = 200)	P
Age, years	55.7 ± 7.6	56.9 ± 8.4	0.14
Gender (M/F)	172/68	145/55	0.85
BMI, kg/m^2^	25.3 ± 3.2	25.5 ± 4.5	0.68
Hypertension, n (%)	126 (52.5)	119 (59.5)	0.14
Diabetes mellitus, n (%)	17 (7.1)	21 (10.5)	0.20
Smoking, n (%)	62 (25.8)	45 (22.6)	0.43
Hypercholesterolemia, n (%)	25 (10.4)	21 (10.5)	0.98
CAD, n (%)	5 (2.1)	10 (5.0)	0.09
paroxysmal/persistent, n (%)	—	109/91 (54.5/45.5)	
LA dimension > 40 mm, n (%)	—	86 (43.0)	
ARB, n (%)	65 (27.1)	83 (41.5)	<0.001
ACE inhibitor, n (%)	15 (6.2)	11 (5.5)	0.74
β-blocker, n (%)	56 (23.3)	88 (44.0)	<0.001
Calcium antagonist, n (%)	71 (29.6)	79 (39.5)	0.03
Diuretic, n (%)	11 (4.6)	32 (16.0)	<0.001
Digoxin, n (%)	0 (0.0)	34 (17.0)	<0.001
Statin, n (%)	41 (17.1)	59 (29.5)	0.002
Aspirin, n (%)	17 (7.1)	85 (42.5)	<0.001
Oral anticoagulant, n (%)	0 (0.0)	38 (19.0)	<0.001
Systemic embolization, n (%)	0 (0.0)	9 (4.5)	0.001

ACE = angiotensin converting enzyme; ARB = angiotensin receptor blocker; BMI = body mass index; CAD = coronary artery disease; LA = left atrium.

**Table 2:** Genotype distribution and allele frequencies of the iNOS promoter in the study population.

	Control (n = 240)	AF (n = 200)	P
Alleles, n (%)			0.167
S (n < 12)	213 (44.4%)	159 (39.8%)	
L (n ≧ 12)	267 (55.6%)	241 (60.2%)	
Genotype, n (%)			0.010
S/S	41 (17.1%)	39 (19.5%)	
L/S	131 (54.6%)	81 (40.5%)	
L/L	68 (28.3%)	80 (40.0%)	
LL carrier	68 (28.3%)	80 (40.0%)	0.010
Non‐LL carrier	172 (71.7%)	120 (60.0%)	

**Table 3:** Clinical characteristics of patients with normal SR and AF at the time of cardiac surgery.

No.	Age (yr)	Sex	CCTTT-repeat number	Underlying cardiac disease			DM	hypertension	LV Ejection Fraction (%)	LAD (mm)	Previous medical history					
				CAD	Operative Indication	Duration of AF (yr)					β blocker	Digitalis	statins	Diuretics	ACE inhibitors or ARB	Calcium channel blockers
**SR**																
1	69	M	14/17	−	AR	−	+	+	71	43	−	−	−	−	−	−
2	61	M	12/12	−	AS+MS	−	−	+	68	47	−	−	+	+	+	−
**AF**																
1	64	F	10/10	−	MS	3	−	−	68	56	−	−	−	+	+	−
2	55	F	10/11	−	AS+MS	6	−	−	74	56	−	+	−	+	+	−
3	48	F	10/11	−	MR	10	−	+	70	59	+	−	−	+	−	−
4	79	M	11/12	−	MR	<1	−	−	59	59	−	−	−	−	−	−
5	62	F	13/14	−	MS	4	−	−	66	50	+	+	−	−	−	−
6	59	F	16/16	−	MS	9	−	−	69	65	−	−	−	−	−	−
7	63	M	16/16	+	MR	8	+	+	44	41	+	−	+	+	+	−

CAD, coronary artery disease; DM, diabetes mellitus; LV, left ventricle; LAD, LA diameter; ARB, angiotensin receptor blocker; ACE, angiotensin converting enzyme; AR, aortic regurgitation; AS, aortic stenosis; MR, mitral regurgitation; MS, mitral stenosis; CABG, coronary artery bypass graft.
