# Supplementary material for: Association evidence of CCTTT repeat polymorphism in the iNOS promoter and the risk of atrial fibrillation in Taiwanese
Source: Sci Rep. 2017 Feb 13;7:42388. doi: 10.1038/srep42388 (PMC5304328; doi:10.1038/srep42388)
Supplement: Supplemental Table S1 [file srep42388-s1.doc]

**Association evidence of CCTTT repeat polymorphism in the iNOS promoter and the risk of atrial fibrillation in Taiwanese**

^1*^Lung-An Hsu, MD, PhD; ^1^Yung-Hsin Yeh, MD; ^1^Wei-Jan Chen, MD, PhD;  ^1^Chi-Tai Kuo, MD; ^2^Feng-Chun Tsai, MD; ^1^Yi-Hsin Chan, MD; ^1^Chun-Li Wang, MD; ^1^Chi-Jen Chang, MD; ^1^Hsin-Yi Tsai, MS

^1^Cardiovascular Division, Chang Gung Memorial Hospital, Chang Gung University College of Medicine, Tao-Yuan, Taiwan; ^2^Division of Cardiac Surgery, Chang Gung Memorial Hospital, Chang Gung University College of Medicine, Tao-Yuan, Taiwan

Supplemental Table S1. Sensitivity analysis for classification of the *iNOS* (CCTTT)n repeats

| Cutoff (n) | Alleles | Control  (n = 240) | AF  (n = 200) | Genotypes, n (%) | Control  (n = 240) | AF  (n = 200) | *P* | Adjusted *P* |
| --- | --- | --- | --- | --- | --- | --- | --- | --- |
| 9 | n<9, S | 2 (0.4%) | 1 (0.2%) | SS + SL | 2 (0.8%) | 1 (0.5%) | 1.000 | 1.00 |
|  | n≧9, L | 478 (99.6%) | 399 (99.8%) | LL | 238 (99.2%) | 199 (99.5%) |  |  |
| 10 | n<10, S | 21 (4.4%) | 19 (4.8%) | SS + SL | 21 (8.8%) | 19 (9.5%) | 0.785 | 1.00 |
|  | n≧10, L | 459 (95.6%) | 381 (95.2%) | LL | 219 (91.2%) | 181 (90.5%) |  |  |
| 11 | n<11, S | 125 (26%) | 96 (24%) | SS + SL | 108 (45.0%) | 76 (38.0%) | 0.138 | 0.3105 |
|  | n≧11, L | 355 (74%) | 304 (76%) | LL | 132 (55.0%) | 124 (62.0%) |  |  |
| 12 | n<12, S | 213 (44.4%) | 159 (39.8%) | SS + SL | 172 (71.7%) | 120 (60.0%) | 0.010* | 0.090* |
|  | n≧12, L | 267 (55.6%) | 241 (60.2%) | LL | 68 (28.3%) | 80 (40.0%) |  |  |
| 13 | n<13, S | 315 (65.6%) | 245 (61.2% ) | SS + SL | 214 (89.2%) | 165 (82.5%) | 0.044 | 0.198 |
|  | n≧13, L | 165 (34.4%) | 155 (38.8%) | LL | 26 (10.8%) | 35 (17.5%) |  |  |
| 14 | n<14, S | 380 (79.2%) | 304 (76.0%) | SS + SL | 231 (96.2%) | 185 (92.5%) | 0.085 | 0.255 |
|  | n≧14, L | 100 (20.8%) | 96 (24%) | LL | 9 (3.8%) | 15 (7.5%) |  |  |
| 15 | n<15, S | 411 (85.6%) | 355 (83.8%) | SS + SL | 235 (97.9%) | 194 (97.0%) | 0.540 | 0.972 |
|  | n≧15, L | 69 (14.4%) | 65 (16.2%) | LL | 5 (2.1%) | 6 (3.0%) |  |  |
| 16 | n<16, S | 430 (89.6%) | 362 (90.5%) | SS + SL | 238 (99.2%) | 198 (99.0%) | 1.000 | 1.000 |
|  | n≧16, L | 50 (10.4%) | 38 (9.5%) | LL | 2 (0.8%) | 2 (1.0%) |  |  |
| 17 | n<17, S | 460 (95.8%) | 383 (95.8%) | SS + SL | 239 (99.6%) | 200 (100.0%) | 1.000 | 1.000 |
|  | n≧17, L | 20 (4.2%) | 17 (4.2%) | LL | 1 (0.4%) | 0 (0.0%) |  |  |
| 18 | n<18, S | 473 (98.5%) | 397 (99.2%) | SS + SL | 240 (100%) | 200 (100%) | - | - |
|  | n≧18, L | 7 (1.5%) | 3 (0.8%) | LL | 0 (0.0%) | 0 (0.0%) |  |  |

Adjusted *P* values were shown with False Discovery Rate corrections
